# mRNA Localization Mechanisms in *Trypanosoma cruzi*

**DOI:** 10.1371/journal.pone.0081375

**Published:** 2013-12-04

**Authors:** Lysangela R. Alves, Eloise P. Guerra-Slompo, Arthur V. de Oliveira, Juliane S. Malgarin, Samuel Goldenberg, Bruno Dallagiovanna

**Affiliations:** Laboratório de Regulação da Expressão Gênica, Instituto Carlos Chagas, Fiocruz-Paraná. Curitiba, Paraná, Brasil; Instituto Butantan, Laboratório Especial de Toxinologia Aplicada, Brazil

## Abstract

Asymmetric mRNA localization is a sophisticated tool for regulating and optimizing protein synthesis and maintaining cell polarity. Molecular mechanisms involved in the regulated localization of transcripts are widespread in higher eukaryotes and fungi, but not in protozoa. Trypanosomes are ancient eukaryotes that branched off early in eukaryote evolution. We hypothesized that these organisms would have basic mechanisms of mRNA localization. FISH assays with probes against transcripts coding for proteins with restricted distributions showed a discrete localization of the mRNAs in the cytoplasm. Moreover, cruzipain mRNA was found inside reservosomes suggesting new unexpected functions for this vacuolar organelle. Individual mRNAs were also mobilized to RNA granules in response to nutritional stress. The cytoplasmic distribution of these transcripts changed with cell differentiation, suggesting that localization mechanisms might be involved in the regulation of stage-specific protein expression. Transfection assays with reporter genes showed that, as in higher eukaryotes, 3′UTRs were responsible for guiding mRNAs to their final location. Our results strongly suggest that *Trypanosoma cruzi* have a core, basic mechanism of mRNA localization. This kind of controlled mRNA transport is ancient, dating back to early eukaryote evolution.

## Introduction

The localization of mRNA and its translation in specific subcellular compartments constitute a posttranscriptional mechanism for regulating gene expression in most eukaryotes [1]. An asymmetric distribution of mRNA is essential for the maintenance of cell polarity, organelle-specific protein expression and the sequestering of proteins in specialized cellular foci [2]. Several studies have indicated that this mechanism is widely distributed in eukaryotic cells [3], [4]. The localization of mRNA involves the interaction of *cis* elements known as zipcodes, generally located in the 3′ untranslated region, with *trans*-acting factors called zipcode binding proteins. The resulting ribonucleoprotein complexes (RNPs) associate with the cytoskeleton and motor proteins, which carry the mRNAs to specific destinations [5].

Such mechanisms have been less studied in lower eukaryotes, but RNA localization has been described in fungi, in which microtubule-mediated RNA transport is essential for rapid polar growth [6]. In yeast, the most extensively studied mechanisms are those involved in the localization of ASH1 mRNA to the bud tip of dividing cells [7]. She2p and She3p proteins are involved in ASH1 transport through binding to the actin cytoskeleton [8].

Trypanosomes branched off early in the evolution of eukaryotes and several species cause diseases with a major impact on public health. Trypanosomatids have unusual biological features, including an absence of typical promoter regions and, hence, transcriptional regulation. Posttranscriptional mechanisms therefore control gene expression in these organisms [9]. The export of mRNA from the nucleus is poorly understood in trypanosomes and has been the subject of intensive research in recent years. Genomic comparisons indicate that the basic components of the RanGTP-dependent RNA pathways are conserved in trypanosomes [10]. RNA-binding proteins (RBPs) involved in various steps of nucleocytoplasmic transport have been characterized in *Trypanosoma cruzi* [11], [12]. Despite the essential nature of posttranscriptional regulation in these lower eukaryotes, no mechanisms for controlling the cytoplasmic localization of specific transcripts have been described in either trypanosomatids or other protozoa. General mRNA localization mechanisms involve aggregation into RNA granules [13], [14]. In conditions of stress, ribonucleoprotein complexes fuse to form mRNA granules, in which transcripts are stored and protected from degradation. Trypanosomes use these structures to compartmentalize ribonucleoprotein complexes in the cytoplasm [15]. However, no specific cytoplasmic localization of transcripts has been described in trypanosomes under physiological conditions.

We investigated the presence of mRNA localization mechanisms in epimastigote forms of *T. cruzi*, which display a marked anterior/posterior polarity. We used FISH to investigate the distribution within the cell of transcripts encoding proteins with specific patterns of cellular expression.

## Materials and Methods

### T. cruzi and T. brucei cultures

Epimastigotes of *T. cruzi* clone Dm28c [16] were grown in liver infusion tryptose (LIT) medium supplemented with 10% heat-inactivated fetal bovine serum at 28°C. Where indicated, Dm28c epimastigotes were subjected to nutritional stress in TAU (triatomine artificial urine) medium containing 190 mM NaCl, 17 mM KCl, 2 mM CaCl_2_, 2 mM MgCl_2_, 0.035% sodium bicarbonate 8 mM phosphate, pH 6.9, at 28°C for 2 hours. Epimastigotes were allowed to differentiate into infectious metacyclic trypomastigotes *in vitro*, as previously described [17]. *T. brucei* strain 29–13 was cultured in SDM-79, as previously described [18].

### Fluorescence *in situ* hybridization (FISH)

FISH assays were carried out with a modified version of a previously described protocol [12], [19]. Briefly, exponentially growing or nutritionally stressed *T. cruzi* epimastigotes, metacyclic trypomastigotes and *T. brucei* procyclic forms were washed three times in PBS (stressed epimastigotes) or PSG (*T. brucei* procyclic forms and *T. cruzi* epimastigotes and metacyclic forms), fixed by incubation with freshly prepared 4% paraformaldehyde for 10 min at room temperature and then washed three times in PBS. Parasites (10^6^/cell per slide) were allowed to adhere to poly-L-lysine-coated microscope slides for 10 minutes at room temperature and the slides were then washed three times with PBS. *T. cruzi* cells were rendered permeable by incubation with 200 mM HCl for 10 minutes at room temperature, whereas *T. brucei* cells were permeabilized by incubation with 0.2% Triton X-100 in PBS for 30 min at room temperature. Cells were then washed five times in RNase-free PBS and all the reagents used for subsequent steps were also RNase-free. Prehybridization was performed in 10 x Denhardt's solution, 4 x SSC, 1 mM EDTA, 35% deionized formamide, 0.5 mg ml ^−1^ tRNA, 40 U ml ^−1^ RNase OUT for 30 min at room temperature. As a control, cells were first treated with either 1 U per 10^6^ cells of RNase-free DNAse I (Promega) for 30 min at room temperature or 100 µg ml ^−1^ boiled RNase A in PBS for 60 min at 37°C. We used β-tubulin, PFR2, cruzipain and oligo d(T)_20_ probes conjugated with Cy-3 or Cy-5 at the 5′ end at a concentration of 50 ng/µl in prehybridization buffer. The cells were heated to 75°C for 5 min and were then allowed to hybridize to the probes at room temperature overnight. The parasites were washed twice with 2 x SSC for 15 min, twice with 1 x SSC for 15 min, and then incubated with 100 ng ml ^−1^ DAPI (Sigma) for 5 min at room temperature. They were mounted on slides in 200 µg ml^−1^ N-propyl gallate and visualized with a Nikon E600 microscope. Images were acquired with the Image Pro program (Media Cybernetics, Bethesda, MD, USA). For plane Z reconstruction for the cruzipain probe, images were acquired with a confocal Leica TCS SP5 AOBS microscope equipped with a 63 x/HCX 1.4 PL Apo lbdBL oil immersion objective. The probes used for the FISH assay are shown in Table S1. The resulting images are from three independent assays, and at least 90% of the cells analysed presented the pattern described in the figure, an average of 100 cells per picture, where three to five different pictures per slide were taken. Relative fluorescence intensity was analyzed using Image J v. 1.47.

### 3′-UTR cloning

The tubulin, PFR2 and GAPDH 3′ untranslated regions were inserted between the *Nhe*I and *Xho*I sites of the pTCDUALuc vector [20]. Probes for the *Renilla* and firefly luciferase mRNAs were labeled with Cy-5 and Cy-3 and used for FISH assays. For cloning of the PFR2 (ID: Tc.CLB.508961.79) and Cruzipain (ID: Tc.CLB.507603.260) 3′-UTRs, we used the following primers: forward (GTAACTCGAGTTTATTGTGGATGTGAC) and reverse (CCAGGCTAGCTAAGGACCAACA), forward (GTAACTCGAGTACTGCTTGTGTGGGTGTGTTTCCTT) and reverse (CCAGGCTAGCGGGCACTCTTTGTTTCTGATGCTG), respectively.

### Cell fractionation and western blot assays

Parasites were treated with lysis buffer (100 mM KCl, 5 mM MgCl_2_, 10 mM Hepes pH 7.0 and 0.5% Nonidet P-40) and centrifuged at 30,000 *x g* for 30 min for separation of cytoplasmic and nuclear/vesicular fractions. For purifying the reservosomal fraction, parasites were lysed by sonication and fractionated by ultracentrifugation on sucrose gradient as described [21]. Reservosomal fraction was disrupted by 5 cycles of freeze-thaw in hypotonic buffer (10 mM HEPES pH 7,4, 10 mM KCl, 1,5 mM MgCl_2_, supplemented with recommended amounts of protease inhibitors PMSF, E-64, EDTA, Aprotinin and Pepstatin-A) and centrifuged at 30,000 *xg* for 30 min for recovering the interior of the organelle. Protein fractions were separated on 15% SDS-PAGE and transferred onto nitrocellulose membrane. After blocking with low-fat milk, membranes were treated with anti-serum raised against TcRBP40 (1∶300), Histone H2A.Z (1∶1000), Cruzipain, 40S ribosomal S7 (1∶500) and 60S ribosomal L26 (1∶500) proteins in PBS and 0,1% Tween, and latter with anti-mouse or -rabbit IgG conjugated with peroxidase for chemiluminescence reaction or DyLight 680 nm for scan on LI-COR's Odyssey.

### RNA isolation and quantitative real-time PCR:

Total, cytoplasmic, intact reservosomal and disrupted reservosomal fractions were submitted to RNA extraction with RNeasy™ kit (Qiagen). Quality check and quantification were performed on Bioanalyzer (Agilent Technologies). RNAs from total, cytoplasmic and intact reservosomal fractions were reverse transcribed with the ImProm-II™ Reverse Transcriptase kit (Promega) using 10 µM of oligo-d(T) primer for 2 h at 42°C. qPCR reactions were performed with SYBR® Green PCR Master Mix (Applied Biosystems) using 1 ng cDNA/20 µL reaction and 5 µM of specific forward and reverse primers for Cruzipain, TcRBP15, TcRBP40, 60S ribosomal protein L9 and RNA polymerase II subunit 9 (Table S1). Program setup was as follows: initial denaturation at 95°C for 15 min and 45 cycles of 95°C for 15 sec, 62 or 64°C for 20 sec and 72°C for 45 sec. Data Analysis were performed in biological triplicates using Pfaffl model [22], where the reference used was RNA PolII amplification data. Error bar indicates standard error between gene and reference samples. Cruzipain and TcRBP15 p-value <0.0035.

## Results

### mRNAs display specific cellular compartmentalization in epimastigote forms

**Figure 1:**
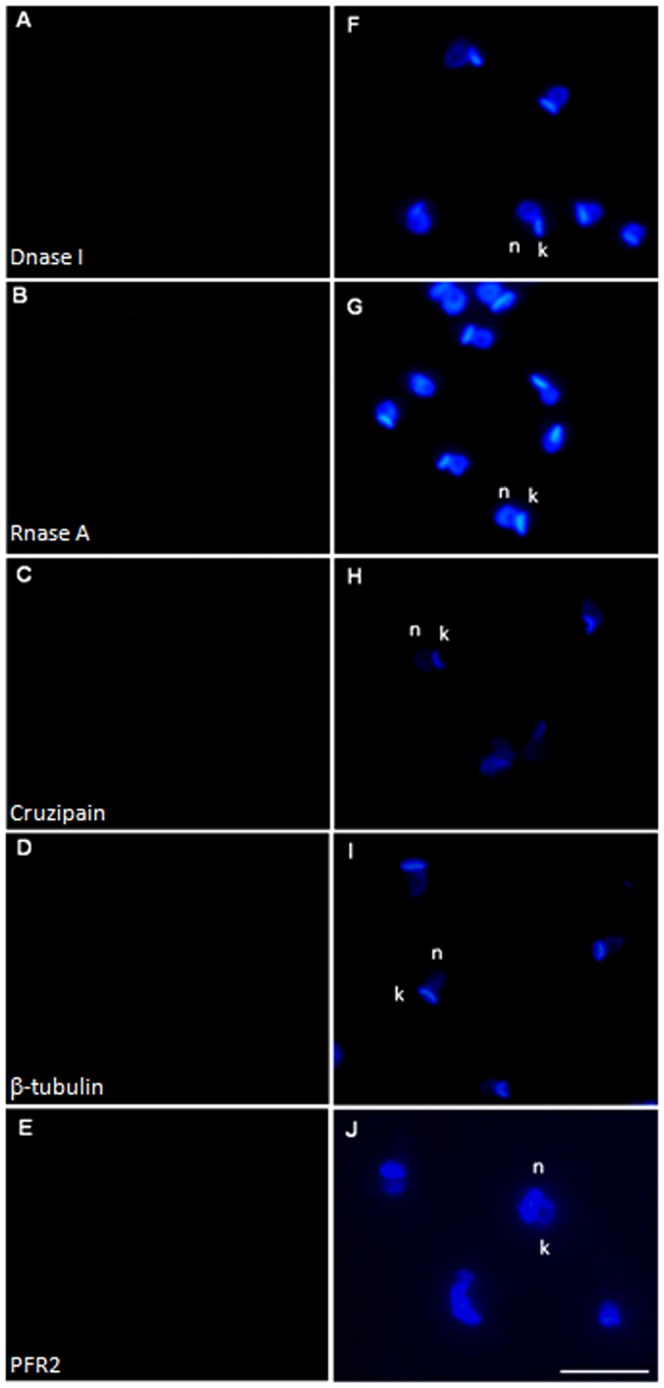
Controls used for FISH validation. A) DNase I treatment before poly-T probe incubation. B) RNase A treatment before poly-T probe incubation. C) Cruzipain sense probes Cy-5 labeled in epimastigotes. D) β-tubulin sense probes Cy-3 labeled in epimastigotes. E) PFR2 sense probes Cy-3 labeled in epimastigotes. F) to J) Merged images, counterstaining with DAPI (blue) was used to identify nuclei (n), kinetoplast (k). Scale bars  = 10 µm.

We used FISH to investigate the subcellular distribution of transcripts for proteins with specific patterns of expression within cells. In all cases, sense probes were used and samples were initially treated with RNase and DNase as negative controls (Figure 1).

**Figure 2:**
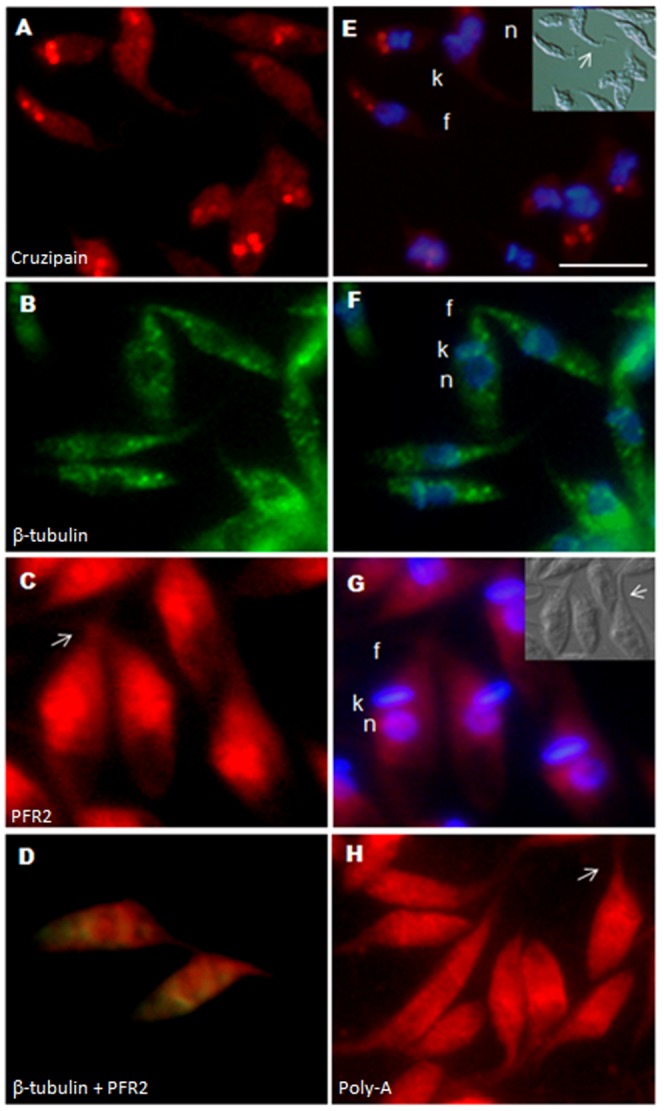
Subcellular localization of *T. cruzi* mRNAs. A) Cruzipain (Cy-5-labeled); B) β-tubulin (Cy-3-labeled); C) PFR2 (Cy-5-labeled); D) merged image of the β-tubulin (Cy-3-labeled) and PFR2 (Cy-5-labeled) probes; E) to G) merged images, counterstaining with DAPI (blue) was used to identify nuclei (n) and kinetoplast (k), flagellum (f); H) Poly-A mRNA (Cy-5-labeled). Differential interference contrast images are shown for identification of the cellular body of the parasite and the flagellum. F) to I) Scale bar  = 10 µm. White arrows indicate the position of the flagellum.

Cruzipain is the major cysteine proteinase of *T. cruzi* and a marker of reservosomes, a vacuolar organelle present in the posterior region of the epimastigote cytoplasm [23]. The cruzipain mRNA was found in granules located at a site resembling the reservosomes containing this proteinase (Figure 2A and E).

β-tubulin is one of the most abundant proteins in epimastigote forms [24]. β-tubulin mRNA tended to be more abundant in the perinuclear region of exponentially growing epimastigotes, in which a granular pattern was observed throughout the cytoplasm (Figure 2B and F).

The paraflagellar rod protein 2 (PFR2) is one of the main components of the paraflagellar rod, a specialized structure that runs along the single anterior flagellum of trypanosomes [25]. PFR2 mRNA in epimastigotes was mostly concentrated at the anterior pole of the cell (Figure 2C and G), proximal to the base of the flagellum, where the encoded protein is localized (Figure S1A).

To confirm the specificity of the localization of the probes used, the β-tubulin and the PFR2 probes were colocalized in the same cell (epimastigote form). In a colocalization assay it is possible to observe the perinuclear pattern of the β-tubulin mRNA while PFR2 is more concentrated at the anterior pole of the parasite (Figure 2D). As a control, we used oligo(d)T probes to determine the overall distribution of transcripts. Oligo(d)T probes showed that mRNAs were uniformly distributed in epimastigote cells (Figure 2H).

The differential localization of these mRNAs was confirmed by measurement of the relative fluorescence intensity of selected areas of the cell. Stronger intensity signals were obtained in the perinuclear region for β-tubulin and in the anterior cytoplasmic region for PFR2 (Figure S1B-D and Table S2).

**Figure 3:**
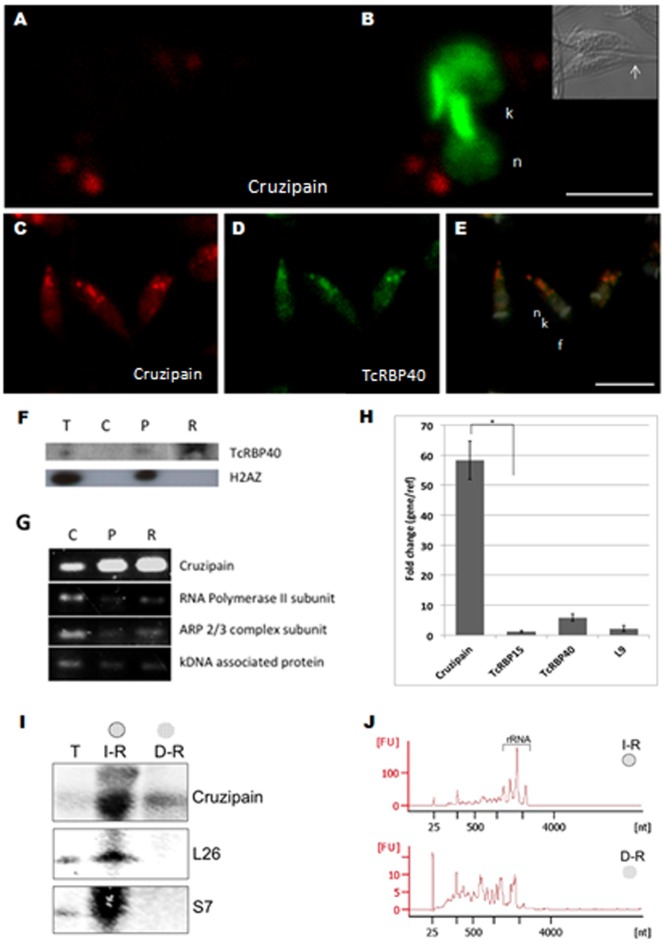
Cruzipain mRNA within reservosomes and colocalization of TcRBP40 protein and cruzipain mRNA. A) Plane Z reconstruction from confocal images obtained with cruzipain probes labeled with Cy-5 in epimastigotes. B) Merged image counterstaining with DAPI (green); Differential interference contrast (DIC) images are shown for identification of the cellular body of the parasite and the flagellum. Scale bar  = 10 µm. White arrows indicate the position of the flagellum. Colocalization of C) cruzipain mRNA labeled with Cy-5 and D) TcRBP40 protein. E) Merged image counterstaining with DAPI (blue) was used to identify the nuclei (n) and kinetoplast (k), flagellum (f). F) Western blot of protein extracts from the same fractions using antibodies against TcRBP40 and Histone H2AZ. G) RT-PCR of RNA obtained from the different cellular fractions of *T. cruzi* epimastigote form, S – soluble cytoplasm, P – pellet, R – reservosome enriched fraction. H) Quantitative PCR for Cruzipain, TcRPB15, TcRBP40 and L9 transcripts enrichment in the reservosome compared to the soluble cytoplasm fractions. The reference used was RNA Pol II and the error bars are indicated. *p-value <0.0035. I) Western blot of total (T), intact (I-R) or disrupted (D-R) reservosomal protein extracts using antibodies against Cruzipain, 40S ribosomal S7 and 60S ribosomal L26 proteins. J) Bioanalyzer's electropherograms of RNAs extracted from intact (I-R) and disrupted (D-R) reservosomal fractions. Peaks corresponding to rRNAs are shown in fraction I-R.

The posterior granular pattern of the cruzipain mRNA suggests the localization of the transcripts in or around reservosomes. Confocal microscopy observations suggested that the cruzipain mRNA was actually located inside the reservosomes (Figure 3A and B). To confirm if the granules observed for the cruzipain mRNA are indeed reservosomes, we performed immunolocalization assays with a TcRBP40 antibody and the cruzipain mRNA probe. TcRBP40 is a *T. cruzi* RNA-binding protein that is localized mainly in the reservosomes [26]. It is possible to observe that the cruzipain mRNA totally colocalizes with the TcRBP40 protein, confirming the reservosome localization for this mRNA (Figure 3 C, D and E). To further confirm the specific localization of cruzipain transcripts we purified reservosomes by cell fractionation as previously described [21], [27]. The identity of the reservosome fraction was shown by western blot of protein extracts using the antibody against TcRBP40. As a control, an antibody against histone H2AZ was used to quantify the possible contamination of this fraction with nuclear proteins or RNA (Figure 3F). RT-PCR analysis of RNA purified from the soluble cytosolic, an insoluble total (including nucleus) and the reservosome protein fractions showed the enrichment of cruzipain and TcRBP40 transcripts compared to control mRNAs, such as RNA pol II and kDNA associated protein (Figure 3G). The transcript levels of cruzipain, TcRBP40 and TcRBP15, a cytoplasmic RNA binding protein, in the reservosome fraction were then quantified by qPCR. For cruzipain, a 60-fold enrichment in the reservosome fraction was observed when compared to the cellular RNA (Figure 3H). This result shows the enrichment of the cruzipain transcript in this organelle. This result opened the possibility of translation inside this organelle. To study the presence of ribosomes we looked for the presence of ribosomal proteins and rRNA. Reservosomes were fractionated followed by membrane disruption to obtain the inside content of the organelle. Western blot assays showed that it was not possible to detect ribosomal proteins inside the vesicles (Figure 3I); however, ribosomal proteins were detected when the integrity of the reservosome was maintained. Cruzipain was detected in the total fraction as well as in the intact and disrupted organelle fractions (Figure 3I). As a complementary approach, the RNA fraction of the intact and the disrupted reservosome was extracted. The Bioanalyzer profile (Figure 3H) showed the presence of rRNA in the intact purified organelle. However, the analysis of the disrupted reservosome showed that rRNA was barely detected (Figure 3H). To further investigate if the cruzipain RNA in the reservosome is being stored or degraded, RNA was purified from the organelle followed by amplification using oligo-d(T) (Figure 3G). In *T. cruzi*, as in yeast, the main pathway of RNA degradation involves deadenylation of the poly-A tail, as no decapping enzyme has been described so far [28], [29]. The cruzipain amplification from the reservosome fraction indicates an intact poly-A tail, suggesting storage rather than degradation. The localization of cruzipain mRNA inside the reservosome is striking, nonetheless the biological role of this observation needs to be further investigated.

### The cytoplasmic distribution of mRNAs changes in response to stress and during parasite differentiation

**Figure 4:**
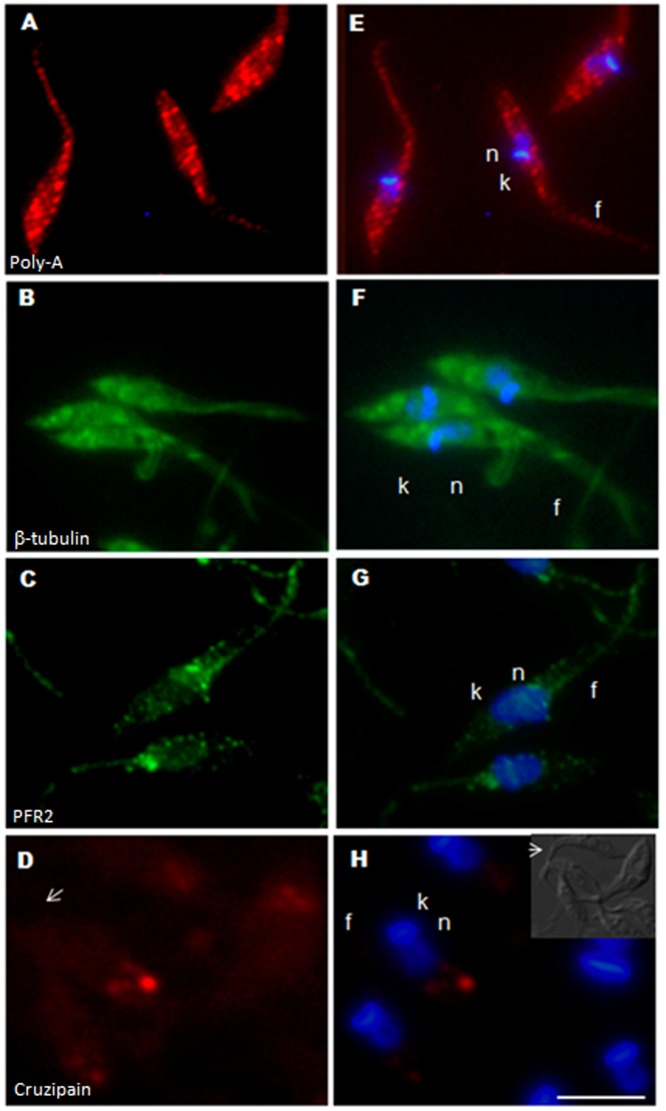
Subcellular localization of specific mRNAs in stressed epimastigotes. A) Poly-A (Cy-5-labeled); B) β-tubulin (Cy-3-labeled); C) PFR2 (Cy-3-labeled); D) Cruzipain (Cy-5-labeled); E) to H) Merged images, counterstaining with DAPI (blue) was used to identify the nuclei (n) and kinetoplast (k), flagellum (f). Differential interference contrast images are shown for identification of the cellular body of the parasite and the flagellum. Scale bar  = 10 µm. White arrows indicate the position of the flagellum.

The regulated localization of mRNAs in granular structures in response to stress has been described as a general mechanism for repressing translation. FISH analysis using poly-T probes against the total population of mRNAs has shown they display a granular distribution during stress in trypanosomatids, suggesting that they are mobilized to form mRNA granules [13]–[15], [30] (Figure 4A and E). However, no localization in RNA granules has been reported for individual transcripts. In epimastigotes subjected to nutritional stress, the distribution of β-tubulin mRNA became more granular, suggesting that it was also mobilized to mRNA granules (Figure 4B and F). This mobilization of transcripts to RNA granules was even more evident for PFR2 mRNA, though some accumulation at the anterior pole remained evident (Figure 4C and G). By contrast, cruzipain transcripts continued to be restricted to the posterior region of the cytoplasm, consistent with the notion that they were localized within the reservosomes (Figure 4D, H and 3).

**Figure 5:**
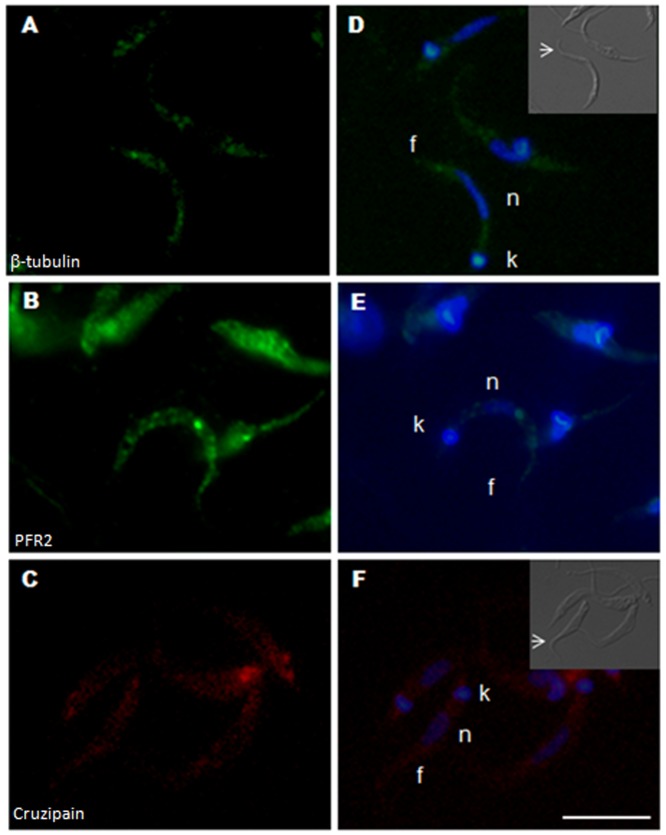
Subcellular localization of specific mRNAs in metacyclic trypomastigotes. A) β-tubulin (Cy-3-labeled); B) PFR2 (Cy-3-labeled); C) Cruzipain (Cy-5-labeled); D) to F) Merged images, counterstaining with DAPI (blue) was used to identify the nuclei (n) and kinetoplast (k), flagellum (f). Differential interference contrast images are shown for identification of the cellular body of the parasite and the flagellum. Scale bar  = 10 µm. White arrows indicate the position of the flagellum.

Within the insect vector, nutritional stress triggers the differentiation of non-infectious epimastigote forms into infectious metacyclic forms. This process can be mimicked *in vitro*, in chemically defined culture conditions [16], [17]. We also investigated the localization of mRNA in infectious metacyclic trypomastigotes. Overall transcription rates are much lower in metacyclic forms than in epimastigotes and the probe signal was therefore much weaker. Nevertheless, the subcellular localization of β-tubulin mRNA in the perinuclear region was clearly maintained (Figure 5A and D). By contrast, the distribution of PFR2 transcripts changed radically with differentiation, from an initially anterior location to a broad distribution throughout the body of the parasite (Figure 5B and E). No cruzipain mRNA was detected in metacyclic forms, consistent with the absence of reservosomes from these infectious parasites (Figure 5C and F).

### Transcript localization in other trypanosomes

**Figure 6:**
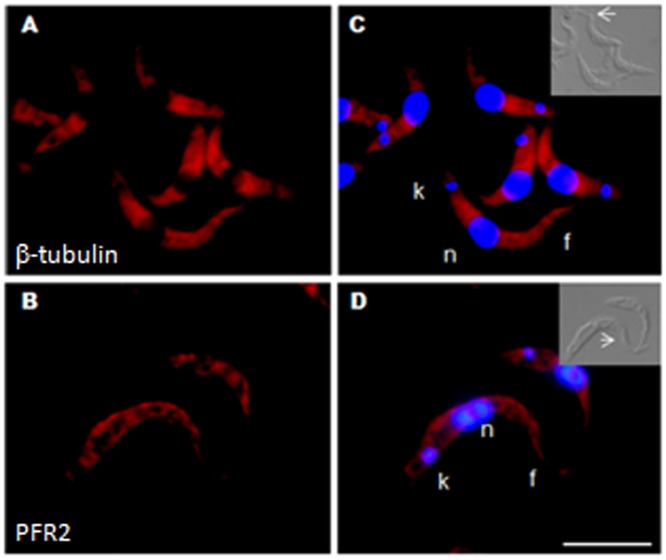
Subcellular localization of specific mRNAs in *T. brucei*. A) β-tubulin probes labeled with Cy-5 in procyclic forms. B) PFR2 probes labeled with Cy-5 in procyclic forms. C) and D) Merged images, counterstaining with DAPI (blue) was used to identify the nuclei (n) and kinetoplast (k), flagellum (f). Differential interference contrast images are shown for identification of the cellular body of the parasite and the flagellum. Scale bar  = 10 µm. White arrows indicate the position of the flagellum.

We then investigated whether these mechanisms of mRNA localization in the cytoplasm also operated in other trypanosomes. We looked for specific mRNA localization, using the same probes for β-tubulin and PFR2, in the insect procyclic forms of *Trypanosoma brucei*. The distribution of β-tubulin mRNA was very similar to that observed in *T. cruzi*, although the perinuclear localization of this mRNA was less obvious than in *T. cruzi* epimastigotes (Figure 6A and C). The TbPFR2 mRNA was also observed in the vicinity of the flagellum, which extends from the posterior to the anterior end in *T. brucei* and is attached to the cell body (Figure 6B and D).

### 3′UTRs direct the subcellular localization of *T. cruzi* mRNAs

**Figure 7:**
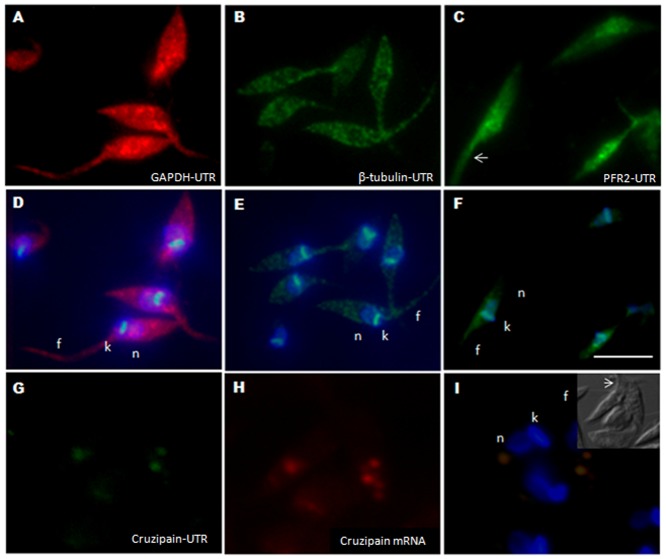
Luciferase mRNA localization in *T. cruzi* with various UTRs. A) Luciferase probes labeled with Cy-5, showing the distribution of UTR-GAPDH as a control. B) Luciferase probes labeled with Cy-5, showing the distribution of UTR-β-tubulin. C) Luciferase probes labeled with Cy-5, showing the distribution of UTR-PFR2. D) to F) Merged images. G) Luciferase probes labeled with Cy-3, showing the distribution of UTR-Cruzipain. H) Cruzipain probes labeled with Cy-5, showing the distribution of cruzipain mRNA. I) Merged images. Counterstaining with DAPI (blue) was used to identify the nuclei (n) and kinetoplast (k), flagellum (f). Differential interference contrast images are shown for identification of the cellular body of the parasite and the flagellum. Scale bar  = 10 µm. White arrows indicate the position of the flagellum.

In other eukaryotes, zipcode elements in the 3′-UTRs of the mRNA are recognized by specific proteins, which direct the mRNA to its subcellular localization. No orthologs of zipcode proteins or putative localization signals in transcripts have been described in trypanosomes. We investigated the possibility that similar elements guide mRNA localization in trypanosomes, by inserting the 3′-UTR containing the complete intergenic region of the β-tubulin, Cruzipain and PFR2 coding genes downstream from the firefly luciferase reporter gene in the pTcDUALuc vector. We transfected *T. cruzi* epimastigotes with these constructs and investigated the cytoplasmic localization of the luciferase mRNA by FISH. The intergenic region of the *gapdh* gene was used as a control. GAPDH transcripts had a diffuse cytoplasmic distribution in epimastigotes (Figure 7A and D). The pattern observed for the reporter transcript with the β-tubulin 3′-UTR was similar to that observed for the endogenous mRNA, although the perinuclear localization was less evident (Figure 7B and E). For constructs containing the PFR2 UTR, the distribution of the luciferase transcripts was predominantly in the posterior region of the cell and virtually indistinguishable from that of PFR2 transcripts in epimastigotes (Figure 7C and F). The results obtained with the cruzipain 3′UTR construct clearly demonstrate the localization of the luciferase transcripts in the reservosomes (Figure 7G), where they colocalize with the cruzipain mRNA (Figure 7H and I). These results suggest that the 3′UTRs of trypanosomes may contain localization elements similar to those present in other organisms.

## Discussion

Specific mRNA localization appears to be a sophisticated tool for regulating and optimizing protein synthesis. Molecular mechanisms for regulating the localization of transcripts are widespread and conserved in higher eukaryotes and fungi. However, no such mechanisms have ever been characterized in protozoa.

Trypanosomes are ancient eukaryotes branching off from the main eukaryote line early in evolution. These unicellular parasites have several unusual features in terms of gene expression and its regulation. The most important of these features is a reliance on posttranscriptional regulation to control differential protein expression during their life cycle and adaptation to different hosts. Several of the cellular forms generated during trypanosome life cycle are highly polarized, with a cytoplasmic compartmentalization of organelles [31]. However, no directed localization of transcripts to cytoplasmic foci has been described. No orthologs of zipcode binding or other RNA-binding proteins involved in mRNA localization have been identified in trypanosome genomes. Nevertheless, given the importance of these mechanisms in posttranscriptional regulation and the importance of posttranscriptional regulation in trypanosomes, we hypothesized that some kind of cytoplasmic localization of transcripts might occur in trypanosomes. FISH analyses with probes for the transcripts of proteins with restricted patterns of expression showed that the corresponding mRNAs were discretely distributed in the cytoplasm, at the same sites as the proteins. The differential localization of ribosomes in epimastigote cells has been recently reported [32]. Accumulation of ribosomes was found in the anterior region of the cell. Moreover, ribosomes were also observed surrounding reservosomes. Though our results do not show a direct relationship between mRNA destination and localized protein translation, the differential localization of transcripts in areas with high densitiy of ribosomes suggest a putative relationship between both biological processes. The cytoplasmic distribution of these transcripts changed with cell differentiation, suggesting that this localization might regulate protein function. Further studies are required to determine whether mRNA transport regulates gene expression.

RNA granules with features reminiscent of P-body-like structures have been described in trypanosomes. These RNPs increase in size and number in response to various types of stress. FISH assays with poly-T probes showed that mRNAs also accumulated in the RNA granules, probably for storage in the cytoplasm [13]–[15], [30]. We demonstrated the mobilization of individual transcripts to RNA granules in response to nutritional stress, suggesting that related mechanisms may be involved in the control of cytoplasmic localization and stress responses.

Cruzipain mRNA was found in the reservosomes, the vacuolar organelles that are located in the posterior region of epimastigote cells [23], [33], [34]. Cruzipain gene family is found in high number in *T. cruzi*. This family presents polymorphic sequences that can generate several different isoforms, which may present different locations and roles in the cell. The presence of cruzipain mRNAs in the cytoplasmic and pellet fractions, besides the reservosome, might reflect the plasticity of this multicopy gene family. This specific localization of mRNAs suggests a more complex metabolic role of this organelle, which was initially described as a simple nutrient reservoir, in *T. cruzi* [33], [34]. RBPs [26] and tRNA-derived small RNAs [19] were recently detected in these organelles. A proteomic analysis of the content of these organelles also revealed the presence of nucleic acid-binding proteins [27]. These observations suggest that reservosomes might play an unexpected role in nucleic acid metabolism. The integrity of the mRNAs localized inside the reservosomes implies that these mRNAs may be functional. However, we did not find evidences of functional ribosomes inside the reservosomes. The observation that there are no ribosomes in the lumen of this organelle suggests a putative mRNA storage or transport role of resevosomes. It was shown that cruzipain is directed to shedding vesicles, which are involved in the establishment of infection [35]. Microvesicles can carry nucleic acids as a mechanism of genetic material transfer between cells [36]. Hence, is tempting to speculate that in *T. cruzi*, the proteins and RNAs directed to the reservosome might be also released from the cell as shedding vesicles for cell-cell communication or to cell-host interaction [37].

The process of mRNA localization involves an interaction between *cis* elements and *trans*-acting factors, generally on the 3′ untranslated region of the transcript [38]. As previously stated, neither *cis* elements nor *trans*-acting factors potentially involved in this process have ever been identified in trypanosomes. Transfection assays with reporter genes showed that, as in higher eukaryotes, the 3′UTRs were responsible for guiding mRNAs to their final location. We can assume that this localization is also dependent on the interaction of protein factors with elements present in the UTR. The absence of genes encoding zipcode-binding proteins from the parasite genome may reflect poor sequence conservation or the existence of other type of RBPs undertaking the role of these specialized proteins.

Thus, although mRNA transport mechanisms seem to be more complex than expected in other lower eukaryotes, such as yeast, our findings point to the existence of a conserved mechanism of specific localization for some mRNAs in basal protozoa. The observations reported here strongly suggest that *T. cruzi* has a core, basic mechanism of mRNA localization, although the sequences involved have not been conserved as in other eukaryotes. Our results suggest that such controlled mRNA transport is ancient, dating back to early eukaryote evolution.

## Supporting Information

Figure S1 **PFR2 and α-FRA colocalization and relative fluorescence intensity of β-tubulin and PFR2 mRNAs in the cell.** A) Colocalization of PFR2 mRNA with FRA protein (flagellar marker). B) Image J integrated density for β-tubulin mRNA. The circles indicate the areas selected for the measurement analysis A- anterior, PN – perinuclear and P - posterior. C) Image J integrated density for PFR2 mRNA. The circles indicate the areas selected for the measurement analysis A- anterior and P - posterior. D) Mean of integrated density plotted in columns for β-tubulin and PFR2, the standard deviation is indicated. T test was applied for significant value *** p≤0.0001. Scale bar  = 10 µm. The α-FRA antibody was used 1:1000 dilution.
(TIF)

Table S1 **List of primers used in FISH and PCR assays.**
(XLSX)

Table S2 **Relative fluorescence intensity of β-tubulin and PFR2 mRNAs in epimastigote cells.**
(XLSX)

## References

[1] Holt CE, Bullock SL (2009) Subcellular mRNA localization in animal cells and why it matters. Science 326: 1212–1216. 19965463 10.1126/science.1176488 PMC3785123

[2] Martin KC, Ephrussi A (2009) mRNA localization: gene expression in the spatial dimension. Cell 136: 719–730. 19239891 10.1016/j.cell.2009.01.044 PMC2819924

[3] Milli S, Macara IG (2009) RNA localization and polarity: from A(PC) to Z(BP). Trends Cell. Biol. 19: 156–164. 19251418 10.1016/j.tcb.2009.02.001 PMC2844668

[4] Lecuyer E, Yoshida H, Parthasarathy N, Alm C, Babak T, et al (2007) Global analysis of mRNA localization reveals a prominent role in organizing cellular architecture and function. Cell 131: 174–187. 17923096 10.1016/j.cell.2007.08.003

[5] Jambhekar A, Derisi JL (2007) Cis-acting determinants of asymmetric, cytoplasmic RNA transport. RNA 13: 625–642. 17449729 10.1261/rna.262607 PMC1852811

[6] Zarnack K, Feldbrugge M (2010) Microtubule-dependent mRNA transport in fungi. Eukaryotic Cell 9: 982–990. 20472693 10.1128/EC.00030-10 PMC2901667

[7] Paquin N, Chartrand P (2008) Local regulation of mRNA translation: new insights from the bud. Trends Cell. Biol. 18: 105–111. 18262421 10.1016/j.tcb.2007.12.004

[8] Long RM, Gu W, Lorimer E, Singer RH, Chartrand P (2000) She2p is a novel RNA-binding protein that recruits the Myo4p-She3p complex to ASH1 mRNA. EMBO J. 19: 6592–6601. 11101531 10.1093/emboj/19.23.6592 PMC305871

[9] Fernández-Moya SM, Estévez AM (2010) Posttranscriptional control and the role of RNA-binding proteins in gene regulation in trypanosomatid protozoan parasites. Wiley Interdiscip. Rev. RNA 1: 34–46. 21956905 10.1002/wrna.6

[10] Serpeloni M, Vidal NM, Goldenberg S, Avila AR, Hoffmann FG (2011a) Comparative genomics of proteins involved in RNA nucleocytoplasmic export. BMC Evol. Biol. 11: 11–17. 21223572 10.1186/1471-2148-11-7 PMC3032688

[11] Cassola A, Frasch AC (2009) An RNA recognition motif mediates the nucleocytoplasmic transport of a trypanosome RNA-binding protein. J Biol. Chem. 284(50): 35015–33028. 19801539 10.1074/jbc.M109.031633 PMC2787363

[12] Serpeloni M, Moraes CB, Muniz JR, Motta MC, Ramos AS, et al (2011b) An essential nuclear protein in trypanosomes is a component of mRNA transcription/export pathway. PLoS One 6(6): e20730. 21687672 10.1371/journal.pone.0020730 PMC3110772

[13] Holetz FB, Correa A, Avila AR, Nakamura CV, Krieger MA, et al (2007) Evidence of P-body-like structures in *Trypanosoma cruzi*. Biochem. Biophys. Res. Commun. 18: 1062–1067. 10.1016/j.bbrc.2007.03.104 17399688

[14] Cassola A, De Gaudenzi JG, Frasch AC (2007) Recruitment of mRNAs to cytoplasmic ribonucleoprotein granules in trypanosomes. Mol. Microbiol. 65: 655–670. 17635187 10.1111/j.1365-2958.2007.05833.x

[15] Cassola A (2011) RNA Granules Living a Post-transcriptional Life: the Trypanosomes' Case. Curr. Chem. Biol. 5: 108–117. 21949551 10.2174/2212796811105020108 PMC3179377

[16] Contreras VT, Araujo-Jorge TC, Bonaldo MC, Thomaz N, Barbosa HS, et al (1988) Biological aspects of the Dm 28c clone of *Trypanosoma cruzi* after metacyclogenesis in chemically defined media. Mem. Inst. Oswaldo Cruz 83: 123–133. 10.1590/s0074-02761988000100016 3074237

[17] Contreras VT, Salles JM, Thomas N, Morel CM, Goldenberg S (1985) In vitro differentiation of *Trypanosoma cruzi* under chemically defined conditions. Mol .Biochem. Parasitol. 16: 315–327. 3903496 10.1016/0166-6851(85)90073-8

[18] Nardelli S, Avila AR, Freund A, Motta MC, Manhães L, et al (2007) Small-subunit rRNA processome proteins are translationally regulated during differentiation of *Trypanosoma cruzi* . Eukaryotic Cell 6: 337–345. 17158738 10.1128/EC.00279-06 PMC1797946

[19] Garcia-Silva MR, Frugier M, Tosar JP, Correa-Dominguez A, Ronalte-Alves L, et al (2010) A population of tRNA-derived small RNAs is actively produced in *Trypanosoma cruzi* and recruited to specific cytoplasmic granules. Mol. Biochem. Parasitol. 171: 64–73. 20156490 10.1016/j.molbiopara.2010.02.003

[20] Araújo PR, Burle-Caldas GA, Silva-Pereira RA, Bartholomeu DC, Darocha WD, et al (2011) Development of a dual reporter system to identify regulatory cis-acting elements in untranslated regions of *Trypanosoma cruzi* mRNAs. Parasitol. Int. 60: 161–169. 21277385 10.1016/j.parint.2011.01.006

[21] Cunha-e-Silva NL, Atella GC, Porto-Carreiro IA, Morgado-Diaz JA, Pereira MG, et al (2002) Isolation and characterization of a reservosome fraction from *Trypanosoma cruzi* . FEMS Microbiol Lett.7 214(1): 7–12. 10.1111/j.1574-6968.2002.tb11317.x 12204365

[22] Pfaffl MW (2001) A new mathematical model for relative quantification in real-time RT-PCR. Nucleic Acids Research 29(9): 2002–2007. 10.1093/nar/29.9.e45 PMC55695 11328886

[23] Cazzulo JJ, Stoka V, Turk V (1997) Cruzipain, the major cysteine proteinase from the protozoan parasite *Trypanosoma cruzi* . Biol. Chem. 378: 1–10. 9049059 10.1515/bchm.1997.378.1.1

[24] de Godoy LMF, Marchini FK, Pavoni DP, Rampazzo RCP, Probst CM, et al (2012) Quantitative Proteomics of *Trypanosoma cruzi* During Metacyclogenesis. Proteomics. doi: 10.1002/pmic.201200078 10.1002/pmic.201200078 22761176

[25] Saborio JL, Manuel Hernandez J, Narayanswami S, Wrightsman R, Palmer E, et al (1989) Isolation and characterization of paraflagellar proteins from *Trypanosoma cruzi* . J. Biol. Chem. 264: 4071–4075. 2645287

[26] Guerra-Slompo EP, Probst CM, Pavoni DP, Goldenberg S, Krieger MA, et al (2012) Molecular characterization of the *Trypanosoma cruzi* specific RNA binding protein TcRBP40 and its associated mRNAs. Biochem. Biophys. Res. Commun. 420(2): 302–307. 22425988 10.1016/j.bbrc.2012.02.154

[27] Sant'Anna C, Nakayasu ES, Pereira MG, Lourenço D, de Souza W, et al (2009) Subcellular proteomics of *Trypanosoma cruzi* reservosomes. Proteomics 9: 1782–1794. 19288526 10.1002/pmic.200800730 PMC2763633

[28] El-Sayed NM, Myler PJ, Bartholomeu DC, Nilsson D, Aggarwal G, et al (2005) The genome sequence of *Trypanosoma cruzi*, etiologic agent of Chagas disease. Science. 15 309(5733): 409–15. 10.1126/science.1112631 16020725

[29] Li CH, Irmer H, Gudjonsdottir-Planck D, Freese S, Salm H, et al (2006) Roles of a *Trypanosoma brucei* 5′-3′ exoribonuclease homolog in mRNA degradation. RNA. 12(12): 2171–86. 17077271 10.1261/rna.291506 PMC1664730

[30] Holetz FB, Alves LR, Probst CM, Dallagiovanna B, Marchini FK, et al (2010) Protein and mRNA content of TcDHH1 containing mRNPs in *Trypanosoma cruzi* . FEBS J. 277(16): 3415–3426. 20629747 10.1111/j.1742-4658.2010.07747.x

[31] De Souza W (1999) A short review on the morphology of *Trypanosoma cruzi*: from 1909 to 1999. Mem. Inst. Oswaldo Cruz 94: 17–36. 10.1590/s0074-02761999000700003 10677689

[32] Girard-Dias W, Alcântara CL, Cunha-e-Silva N, de Souza W, Miranda K (2012) On the ultrastructural organization of *Trypanosoma cruzi* using cryopreparation methods and electron tomography. Histochem Cell Biol. 138(6): 821–31. 22872316 10.1007/s00418-012-1002-8

[33] Figueiredo RC, Rosa DS, Soares MJ (2000) Differentiation of *Trypanosoma cruzi* epimastigotes: metacyclogenesis and adhesion to substrate are triggered by nutritional stress. J. Parasitol. 86: 1213–1218. 11191893 10.1645/0022-3395(2000)086[1213:DOTCEM]2.0.CO;2

[34] Soares MJ, De Souza W (1988) Cytoplasmic organelles of trypanosomatids: a cytochemical and stereological study. J Submicrosc Cytol. Pathol. 20(2): 349–361. 3135113

[35] Aparicio IM, Scharfstein J, Lima AP (2004) A new cruzipain-mediated pathway of human cell invasion by *Trypanosoma cruzi* requires trypomastigote membranes. Infect. Immun., 72: , 5892–5902. 10.1128/IAI.72.10.5892-5902.2004 PMC517595 15385491

[36] Valadi H, Ekstrom K, Bossios A, Sjostrand M, Lee JJ, Lotvall JO (2007) Exosome-mediated transfer of mRNAs and microRNAs is a novel mechanism of genetic exchange between cells. Nat. Cell. Biol., 9: , 654–659. 10.1038/ncb1596 17486113

[37] Torrecilhas AC, Schumacher RI, Alves MJ, Colli W (2012) Vesicles as carriers of virulence factors in parasitic protozoan diseases. Microbes Infect. 4(15): 1465–74. 10.1016/j.micinf.2012.07.008 22892602

[38] Giorgi C, Moore MJ (2007) The nuclear nurture and cytoplasmic nature of localized mRNPs. Cell Dev. Biol. 18: 186–193. 10.1016/j.semcdb.2007.01.002 17459736

