# Supplementary figures and images for: mRNA Localization Mechanisms in Trypanosoma cruzi
Source: PLoS One. 2013 Dec 4;8(12):e81375. doi: 10.1371/journal.pone.0081375 (PMC3852752; doi:10.1371/journal.pone.0081375)

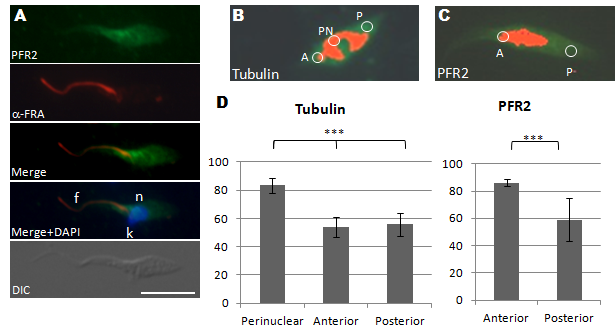

Supplement: Figure S1 — PFR2 and α-FRA colocalization and relative fluorescence intensity of β-tubulin and PFR2 mRNAs in the cell. A) Colocalization of PFR2 mRNA with FRA protein (flagellar marker). B) Image J integrated density for β-tubulin mRNA. The circles indicate the areas selected for the measurement analysis A- anterior, PN – perinuclear and P - posterior. C) Image J integrated density for PFR2 mRNA. The circles indicate the areas selected for the measurement analysis A- anterior and P - posterior. D) Mean of integrated density plotted in columns for β-tubulin and PFR2, the standard deviation is indicated. T test was applied for significant value *** p≤0.0001. Scale bar = 10 µm. The α-FRA antibody was used 1:1000 dilution. (TIF) [file pone.0081375.s001.tif]
